# The Relationship Between Attentional Bias, Anxiety Sensitivity, and Depression and Anxiety Symptoms: Evidence From the COVID-19 Pandemic in China

**DOI:** 10.3389/fpubh.2022.832819

**Published:** 2022-02-08

**Authors:** Shiyi Li, Xiao Li

**Affiliations:** ^1^Academy of Psychology and Behavior, Tianjin Normal University, Tianjin, China; ^2^Faculty of Psychology, Tianjin Normal University, Tianjin, China; ^3^Tianjin Social Science Laboratory of Students' Mental Development and Learning, Tianjin, China

**Keywords:** anxiety sensitivity, physical concerns, cognitive concerns, mental health, attention bias

## Abstract

**Background:**

The COVID-19 pandemic has led to observed increases in reported mental health issues, such as depression and anxiety symptoms. There is evidence attentional bias is associated with depression and anxiety, and it has been further suggested that anxiety sensitivity has a role in both the development and maintenance of depression and anxiety symptoms. Understanding these relationships may help inform preventative interventions for those at risk of mental health concerns. The present study explores the role of anxiety sensitivity, specifically physical and cognitive concerns, as a potential mediator of the relationship between attentional bias with depression and anxiety symptoms.

**Method:**

Participants (*n* = 460) were recruited from the general population in China, and completed an online survey between February and March, 2020 which included the Attention to Positive and Negative Information Scale (APNI), Anxiety Sensitivity Index-3 (ASI-3) and Depression, Anxiety and Stress Scale (DASS-21). After exploring the correlations between the measures, mediation analysis was performed to explore the role of anxiety sensitivity (physical and cognitive subscales) in the relationship between attentional bias and depression and anxiety (as measured by the DASS-21).

**Results:**

The results indicated that negative attention bias was significantly positively correlated with physical and cognitive concerns, physical and cognitive concerns were significantly positively correlated with depression and anxiety, and negative attention bias was significantly positively correlated with depression and anxiety (all *ps* < 0.001). Physical and cognitive anxiety sensitivity mediated the relationship between negative attention bias and both anxiety and depression symptoms.

**Conclusion:**

Negative bias was associated with levels of anxiety and depression, and physical and cognitive anxiety sensitivity mediated associations between negative bias and anxiety and depression symptoms. The study provides theoretical support for intervention and guidance on individual mental health during the pandemic, and helps individuals increase their concern to negative emotions.

## Introduction

Public health issues such as COVID-19 will not only have a huge impact on social production, life and economic conditions, but also affect the physical and mental health of the public. COVID-19 is sudden, because of its fast-spreading speed, wide range, and strong infectivity, it seriously threatens the safety of human life, and has adverse health effects (1). On January 30, 2020, COVID-19 was listed by the World Health Organization (WHO) as a public health emergency of international concern. At present, COVID-19 has had an extraordinary threat on all aspects of individual life, such as safety, health, and wellbeing. The effects also extend to mental health, with associated effects on anxiety, depression, panic and other negative emotions (2). Anxiety and depression are significant indicators of poor mental health (3). Empirical research suggests that mental health disorder, especially negative emotions such as anxiety and depression, are very common, which not only reduces life satisfaction, but also impairs life functioning (4–6).

Studies have suggested that cognition and emotion have interactions at the functional and neurological levels, which together constitute the basis of behavior (7–9). Cognition is a necessary condition for emotion generation. Attention, as an early stage of cognitive processing, will affect individual emotional experience.

Attentional bias is an automatic and uncontrollable unconscious tendency, which refers to prioritizing certain types of stimuli to increase our ability to process this information (10). A number of studies have suggested (11, 12) that anxiety and depression are associated with increased attention allocation to negative stimuli compared to neutral stimuli (i.e., an attentional negativity bias). Attentional bias can be measured both objectively and subjectively. Objective measurement refers to the research conducted from the perspective of behavioral cognition through experimentation (13–15). It is usually inferred by measuring the tendency to pay attention to one type of stimulus over another, such as smoking-related stimuli and neutral control stimuli (16). While subjective measurement meanwhile is typically collected through self-report measures such as the attention to positive and negative information scale which has shown good reliability and validity (17–20).

The cognitive theory of anxiety suggests that attention bias plays an essential role in the maintenance of anxiety (21). Some studies indicated that attention bias is closely relevant to anxiety and depression (22). For example, Joormann and Gotlib found that depressed individuals tend to pay attention to negative material and avoid positive material (23). Koster et al. found that individuals with anxiety attend toward threatening images by using images (threatening images and neutral images) as experimental materials to test the responses of subjects (24). EEG study indicates that negativity biases produce hyper-activation of fear circuits during non-conscious processing of anxiety and conscious processing of depression (25).

However, previous studies have explored the relationship between attentional bias and anxiety and depression (26), but there is a lack of further research on the mediating variables between attentional bias and anxiety and depression. Since the COVID-19, there have been a large number of studies exploring the mental health of people, studying the psychological conditions of different groups, focusing on psychological interventions, etc. (27, 28), but few studies have explored their anxiety from the cognitive level of individuals. Therefore, this study introduced another variable, anxiety sensitivity, to study the indirect effects of attentional bias on anxiety and depression.

Anxiety sensitivity (AS) refers to the fear of anxiety and sensations related to anxiety (29). Individuals with high AS tended to experience various negative emotions (30). AS is a key cognitive factor in the generation and maintenance of anxiety and depression, which can theoretically increase the risk of anxious and depressive psychopathology (31). Studies have found that individuals with high AS have an attention bias toward threat stimuli, which may trigger deeper negative emotions, which in turn deepens their attention to threat stimuli (32). Most research has focused on AS as a whole, and few studies have specifically explored its specific dimensions. AS has three dimensions: cognitive, physical and social concerns (33). Cognitive concern reflect fear of cognitive dyscontrol, physical concern reflect the fear of physical sensations accompanying anxiety, and social concern reflect the fear that an observable anxiety response will lead to social exclusion (34). Many studies have suggested that physical and cognitive mediate the relationship, but social concern was not found to be a significant mediator (35). Guo et al. (36) found that the mediating effect of social concern on anxiety and attentional control is not significant. But the study did not explore the mediating role of different dimensions of anxiety sensitivity between attention bias and other negative emotions.

Previous literature has not been clear on the relationship between attention bias, AS, anxiety and depression. To further the existing literature and integrating existing research results, the study examined the influence of negative bias on anxiety and depression, and further investigated the mediating role of physical and cognitive concerns. Furthermore, the study also investigated the mediating role of social concerns. Based on the above review, the following hypotheses for this study were: negative attention bias would be associated with anxiety and depression symptoms; physical and cognitive AS would mediate relations between negative attention bias and both anxiety and depression symptoms. This study has potential significance for understanding how attentional processing is related to anxiety sensitivity, and then lead to depression and anxiety symptoms during the COVID-19 pandemic. This understanding might be used to support public health messaging, and identify people at risk of poorer mental health during public health emergencies.

## Methods

### Participants and Procedure

The study conducted a cross-sectional internet-based survey of Chinese adults from February to March 2020. This study used the widely popular Chinese social media application “WeChat” to invite participants (37). WeChat has location-based online communities. We arranged for WeChat community moderators from a large city in central China (within Henan province) to invite their residents through the app. It also includes some participants from other provinces, including Beijing, Sichuan, Zhejiang, etc. The present study used the Survey Star online questionnaire. Electronic consent of all participants was obtained in this study. Before obtaining participant consent, they were informed that the survey was anonymous and confidential. And inform the participants that the purpose of this survey is to investigate your mental health, the results of the survey are only used for statistics and not for other purposes. All procedures in this study conformed to the ethical standards of the Chinese Psychological Association (https://www.cpsbeijing.org/) and are in line with the 1964 Helsinki Declaration and subsequent amendments or similar ethical standards. The study was approved by the Tianjin Normal University ethics committee (XL2020-21).

A total of 529 people participated and completed all scales. All the participants had normal vision or corrected vision, without any history of mental disorder. Survey Star provides a feature that prevents participants from answering the questionnaire multiple times, and it prompts participants to complete skipped items, so there is no missing data. We removed participants who were considered to not have given the study proper attention by either providing the same response to all items, or who completed the full questionnaire battery in <180 s. A total of 460 valid questionnaires were retained, with an effective rate of 86.96%. The average age of the participants was 25.38 years (*SD* = 8.73). There were 154 male participants (33.5%) and 306 female participants (66.5%).

### Measures

#### The Attention to Positive and Negative Information Scale (APNI)

The APNI was developed by Noguchi et al. to measure attention bias toward positive or negative information (38). Lv et al. adapted a Chinese scale version, with a total of 30 items, divided into two dimensions: positive attention bias (19 items in total) and negative attention bias (11 items in total) (20). Items are rated on a 5-point Likert scale from 1 (“strongly disagree”) to 5 (“strongly agree”). In this study, the negative attention bias dimension was used to measure attention bias toward negative information. Internal consistency for the sample was 0.878.

#### Anxiety Sensitivity Index-3 (ASI-3)

The ASI-3 was developed by Taylor et al., mainly used to measure fear of anxiety-related symptoms based on the belief that they may have harmful personal consequences (33). The scale consists of three dimensions of physical, cognitive and social concerns. Each dimension has six items, for a total of 18 items. Items are rated on a 5-point Likert scale from 0 (“very little”) to 4 (“very much”) (39). Internal consistency for the present sample was 0.940 for the physical concern subscale, 0.924 for the cognitive concern subscale, and 0.888 for the cognitive concern subscale.

#### Depression, Anxiety, and Stress Scale (DASS-21)

The DASS-21 was initially developed by Lovibond et al. and the Chinese version was developed by Gong et al. (3, 40). The questionnaire has good reliability and validity among Chinese adults, with a wide range of applicability (41). Twenty-one items are rated over the past week on a 4-point Likert scale from 0 (“not applicable”) to 3 (“very applicable”); higher scores reflect greater symptoms. The anxiety and depression sub-scales were used in this study, with Internal consistency of 0.890 and 0.917, respectively.

### Data Analysis

Statistical analyses were conducted using the SPSS (v. 22.0 for Windows; IBM Corporation), the data were analyzed for descriptive statistical analysis, Pearson correlations and regression analysis. The study used deviation-corrected percentile bootstrapping to test the mediating effect. The mediation analyses were conducted using the Hayes Process Macro for SPSS with 95% bias corrected confidence interval (CI) based on 5,000 bootstrap samples (42). The mediating effect exists if the confidence interval does not include 0. According to the hypothesis of this research negative attention bias would be associated with anxiety and depression symptoms; physical and cognitive AS would mediate relations between negative attention bias and both anxiety and depression symptoms. The study have run two analyses, one without the social concern and one with to check for the effect.

## Results

### Descriptive Statistics and Correlation Results

In order to explore relationships between negative attention bias and physical and cognitive concerns, anxiety and depression symptoms, Pearson correlations were conducted, and results are shown in Table 1. Specifically, negative attention bias was significantly positively correlated with physical concern (*r* = 0.408, *p* < 0.001) and cognitive concern (*r* = 0.449, *p* < 0.001), physical concern (*r* = 0.731, *p* < 0.001) and cognitive concern (*r* = 0.747, *p* < 0.001) were significantly positively correlated with depression, physical concern (*r* = 0.783, *p* < 0.001), and cognitive concern were significantly positively correlated with anxiety, and negative attention bias was significantly positively correlated with depression (*r* = 0.399, *p* < 0.001) and anxiety (*r* = 0.431, *p* < 0.001).

**Table 1:** Mean, standard deviation, and correlation coefficient of each variable.

	**1**	**2**	**3**	**4**	**5**
1 APNI negative	–^**^				
2 DASS-21 depression	0.399^***^	–^**^			
3 DASS-21 anxiety	0.431^***^	0.876^***^	–^**^		
4 ASI-3 physical	0.408^***^	0.731^***^	0.783^***^	–^**^	
5 ASI-3 cognitive	0.449^***^	0.747^***^	0.772^***^	0.895^***^	–
*M*	35.18	4.68	5.05	5.90	6.53
*SD*	7.08	4.85	4.73	6.00	5.93

*N = 460; ASI-3, Anxiety Sensitivity Index-3; DASS-21, Depression Anxiety Stress Scale-21; APNI, Attention to Positive and Negative Information Scale*.

^*^p < 0.05,

** *p < 0.01*,

*** *p < 0.001*.

### Mediation Models

With negative attention bias as independent variable, cognitive and physical concerns as mediating variables, anxiety and depression as dependent variables separately, multiple mediation analysis were conducted. The results indicate that negative attention bias was significantly associated with depression and anxiety symptoms (β = 0.078, *p* = 0.022; β = 0.103, *p* = 0.001, respectively). Physical and cognitive concerns were significantly associated with depression (β = 0.304, *p* < 0.001; β = 0.444, *p* < 0.001, respectively), and physical and cognitive concerns were associated with anxiety (β = 0.450, *p* < 0.001; β = 0.327, *p* < 0.001, respectively). Results are reported in Table 2. From the model, negative attention bias not only directly predicted depression and anxiety, but also indirectly predicted depression and anxiety through physical and cognitive concerns.

**Table 2:** Regression analysis of variable relationships in models.

**Outcome variable**	**Predictor variables**	***R***	***R^2^***	***F***	***β***	***t***
		0.440	0.193	36.382^***^		
ASI-3 physical	Sex				−0.157	−3.696^***^
	Age				−0.034	−0.797
	APNI negative				0.391	9.160^***^
		0.484	0.235	46.564^***^		
ASI-3 cognitive	Sex				−0.127	−3.070^**^
	Age				−0.123	−2.950^**^
	APNI negative				0.420	10.115^***^
		0.763	0.583	126.826^***^		
DASS-21 depression	Sex				0.006	0.192
	Age				0.017	0.527
	APNI negative				0.078	2.304^*^
	ASI-3 physical				0.304	4.396^***^
	ASI-3 cognitive				0.444	6.252^***^
		0.804	0.647	166.429^***^		
DASS-21 anxiety	Sex				−0.002	−0.066
	Age				0.016	0.539
	APNI negative				0.103	3.292^*^
	ASI-3 physical				0.450	7.062^***^
	ASI-3 cognitive				0.327	5.000^***^

*All variables were standardized. ASI-3, Anxiety Sensitivity Index-3; DASS-21, Depression Anxiety Stress Scale-21; APNI, Attention to Positive and Negative Information Scale*.

* *p < 0.05*,

** *p < 0.01*,

*** *p < 0.001*.

**Figure 1:**
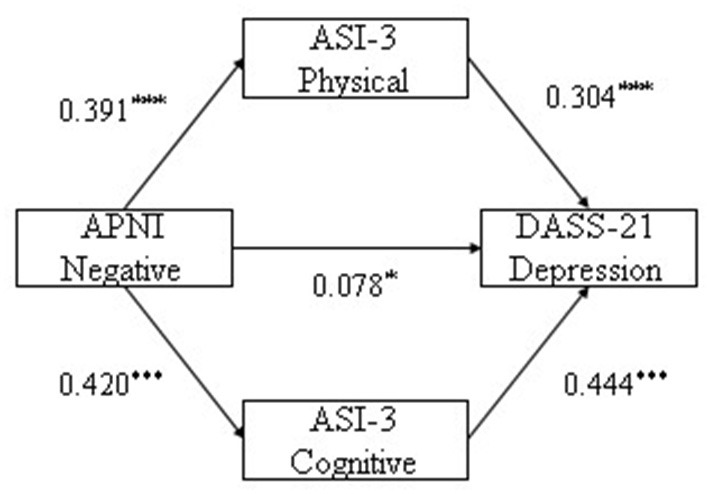
The mediating pathway of physical and cognitive concerns in negative bias influence depression. *p < 0.05, **p < 0.01, ***p < 0.001.

The study analyzed the mediating effect of physical and cognitive concerns between attention bias and depression severity. Results suggested that the indirect effect of physical concern on the influence of negative attention bias on depression was 0.081. Its bootstrapped 95% confidence interval for the indirect effect of “negative attention bias → physical concern → depression” was (0.044, 0.126). The 95% confidence interval did not contain 0, indicating that physical concern had a significant mediating effect. The indirect effect of cognitive concern on the influence of negative bias on depression was 0.128, and the bootstrapped 95% confidence interval for the indirect effect of “negative attention bias → cognitive concern → depression” was (0.083, 0.179).The 95% confidence interval did not contain 0, indicating that cognitive concern had a significant mediating effect (see Table 3 and Figure 1). The finding indicates that physical concern and cognitive concern have significant mediating effects between negative attention bias and depression.

**Table 3:** Analysis of the mediating effect of physical concern and cognitive concern on negative bias affecting depression.

	**Effect**	**BootSE**	**BootLLCI**	**BootULCI**
Total	0.209	0.027	0.158	0.264
ASI-3 physical	0.081	0.021	0.044	0.126
ASI-3 cognitive	0.128	0.025	0.083	0.179

*ASI-3, Anxiety Sensitivity Index-3*.

BootSE, BootLLCI, and BootULCI refer to the standard error, lower limit and upper limit of the 95% confidence intervals of the indirect effects estimated by the percentile bootstrap method corrected by deviation, respectively, as follows.

**Figure 2:**
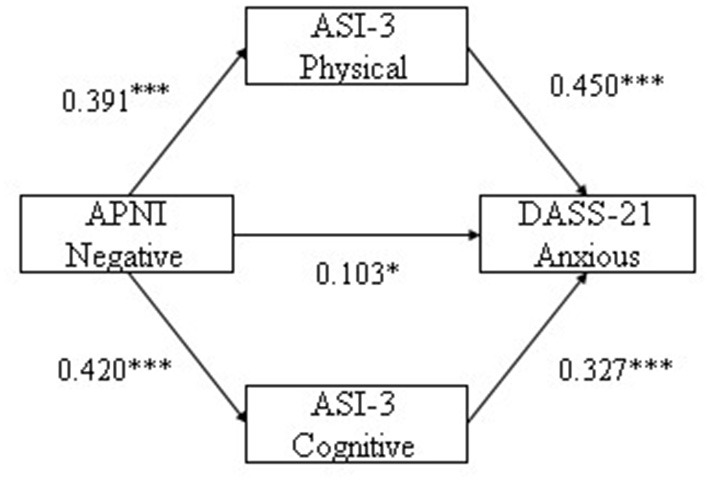
The mediating pathway of physical and cognitive concerns in negative bias influence anxious. *p < 0.05, ***p < 0.001.

Next the study analyzed the mediating effect of physical and cognitive concerns between negative attention bias and anxiety. Results indicated that the indirect effect of physical concern on the influence of negative attention bias on anxiety was 0.117, and its bootstrapped 95% confidence interval for the indirect effect of “negative attention bias → physical concern → anxiety” was (0.079, 0.163). The 95% confidence interval did not contain 0, indicating that physical concern had a significant mediating effect. The indirect effect of cognitive concern on the influence between negative bias on anxiety was 0.092, and the bootstrapped 95% confidence interval for the indirect effect of “negative attention bias → cognitive concern → anxiety” was (0.054, 0.136). The 95% confidence interval did not contain 0, indicating that cognitive concern had a significant mediating effect (see Table 4 and Figure 2). The finding indicates that physical concern and cognitive concern have significant mediating effects between negative attention bias and anxiety.

**Table 4:** Analysis of the mediating effect of physical concern and cognitive concern on negative bias affecting anxiety.

	**Effect**	**BootSE**	**BootLLCI**	**BootULCI**
Total	0.209	0.027	0.157	0.264
ASI-3 physical	0.117	0.022	0.079	0.163
ASI-3 cognitive	0.092	0.021	0.054	0.136

*ASI-3, Anxiety Sensitivity Index-3*.

In addition, the study also explored the mediating role of physical concern, cognitive concern, and social concern between negative bias and depression and anxiety, respectively. The results indicated that the regression coefficient of social concern was not significant (see Table 5), and the bootstrapped 95% confidence interval for the indirect effect of “negative attention bias → social concern → depression” was (−0.068, 0.001) (see Table 6). The 95% confidence interval of each parth contained 0. The bootstrapped 95% confidence interval for the indirect effect of “negative attention bias → social concern → anxiety” was (−0.034, 0.030) (see Table 7). The 95% confidence interval of each parth contained 0. These indicated the mediating effect of social concern was not significant.

**Table 5:** Regression analysis of variable relationships in models (physical, cognitive, and social concerns as mediating variables).

**Outcome variable**	**Predictor variables**	***R***	***R^2^***	***F***	**β**	***t***
		0.440	0.193	36.382^***^		
ASI-3 physical	Sex				−0.157	−3.696^***^
	Age				−0.034	−0.797
	APNI negative				0.391	9.160^***^
		0.484	0.235	46.564^***^		
ASI-3 cognitive	Sex				−0.127	−3.070^**^
	Age				−0.123	−2.950^**^
	APNI negative				0.420	10.115^***^
		0.487	0.237	47.196^***^		
ASI-3 social	Sex				−0.075	−1.813
	Age				−0.153	−3.676^**^
	APNI negative				0.426	10.269^***^
		0.765	0.586	106.827^***^		
DASS-21 depression	Sex				0.010	0.325
	Age				0.009	0.293
	APNI negative				0.087	2.549^*^
	ASI-3 physical				0.323	4.627^***^
	ASI-3 cognitive				0.521	6.347^***^
	ASI-3 social				−0.114	−1.853
		0.804	0.647	138.392^***^		
DASS-21 anxiety	Sex				−0.002	−0.057
	Age				0.015	0.520
	APNI negative				0.104	3.272^*^
	ASI-3 physical				0.451	7.000^***^
	ASI-3 cognitive				0.331	4.369^***^
	ASI-3 social				−0.006	−0.114

* *p < 0.05*,

** *p < 0.01*,

*** *p < 0.001*.

**Table 6:** Analysis of the mediating effect of physical, cognitive, and social concerns on negative bias affecting depression.

	**Effect**	**BootSE**	**BootLLCI**	**BootULCI**
Total	0.203	0.027	0.151	0.258
ASI-3 physical	0.082	0.021	0.048	0.132
ASI-3 cognitive	0.150	0.029	0.096	0.208
ASI-3 social	−0.033	0.017	−0.068	0.001

*ASI-3, Anxiety Sensitivity Index-3*.

**Table 7:** Analysis of the mediating effect of physical, cognitive and social concerns on negative bias affecting anxiety.

	**Effect**	**BootSE**	**BootLLCI**	**BootULCI**
Total	0.209	0.028	0.157	0.265
ASI-3 physical	0.118	0.022	0.078	0.165
ASI-3 cognitive	0.093	0.024	0.048	0.143
ASI-3 social	−0.002	0.016	−0.034	0.030

*ASI-3, Anxiety Sensitivity Index-3*.

## Discussion

Since December 2019, the COVID-19 has affected social production and personal life to varying degrees. With the development of society and economy, in the face of public emergencies, the public's ability to protect against risks economically has increased, but the psychological impact cannot be ignored. Therefore, more and more scholars have begun to explore the public's psychological conditions and risk perceptions behind public events from a psychological perspective (32). In February 2020, Guo et al. (43) surveyed the mental health status of 26,000 Chinese people through an online questionnaire, and found that 33% of the participants had a certain degree of depression, and 22.4% of the participants had obvious anxiety. During the pandemic, the incidence of public anxiety and depression has increased (44).

The present study focus on the relationship between cognition and emotion, especially explored the relationship between negative attention bias and anxiety and depression through physical and cognitive concerns (45). The results indicated that negative bias was significantly positively correlated with anxiety and depression, suggesting that negative bias is a potential influencing factor of anxiety and depression, which would induce negative emotion and have a negative influence on mental health development. During the pandemic, people will receive a lot of news about the COVID-19. Compared with individuals who pay more attention to positive information related to the pandemic, individuals who are biased toward negative news will have more negative emotions such as anxiety or depression. From the perspective of cognition, different cognitive styles will lead to differences in attention bias and affect individual emotional health to varying degrees, thus enhancing negative emotions such as anxiety and depression (46). This may also be one of the reasons why different individuals experience different anxiety and depression in the same context of the pandemic.

Negative attention bias and AS subscales were significantly positively correlated, indicating that when individuals have attention bias to negative information, their worries and fears about their own physical feelings and cognitions increase. The onion model of cognition suggests that cognitive processes and personality factors are interrelated and interact with each other (47). Consistent with previous studies, individual cognitive differences, such as attention bias to negative information, are reasons leading to high AS (27). During the pandemic, some individuals may pay too much attention to the negative information about the pandemic rather than the positive information. As shown in the results of this study, on the one hand, the attentional bias toward negative information will cause individuals to have stronger fears of their own physical sensations (such as sweating, shaking, etc.); on the other hand, it will also cause individuals to have a fear of cognitive dissonance.

According to correlation and regression results excessive worry about physiology and cognition were associated with negative emotions. As cognitive susceptibility factors of mental disorders, AS and its subscales are not only considered as risk factors leading to anxiety disorders, but also as important influencing factors of anxiety and depression (48). They are malleable and easy to be evaluated (49). In view of this, people with high AS may have a low tolerance for emotions such as anxiety, whereas individuals with low AS may have high tolerance (50). Previous studies have also suggested that there is a positive correlation between AS and negative emotions, and individuals with high AS tend to experience various negative emotional states (51). It can be inferred that during the pandemic, individuals with high AS tend to experience more negative emotions such as anxiety or depression. Results of the present study suggest that negative bias may affect anxiety and depression through the mediating effects of physical concern and cognitive concern, respectively. When an individual has attention bias toward negative information, the individual will have a fear of impaired cognitive control, and the individual's cognitive concern will further increase their negative emotional experiences such as anxiety and depression. Researchers believe that cognitive attention will trigger one's negative emotions by amplifying anxiety symptoms, thereby driving the relationship between AS and anxiety and depression (52). This is consistent with results of previous studies (53). Prior work has suggested that cognitive AS can increase anxiety and depression. Individuals with high cognitive concern believe that their symptoms, such as attentional decline or psychological incompetence will cause individuals to feel more uncomfortable, thus further enhancing negative emotional experience (35). The current research lacks evidence that social concern has mediating effect between negative bias and depression and anxiety. It indicates that, individuals pay less attention to society than their physical and cognitive concerns during the pandemic, that is, external evaluations may not trigger individual fear. It is consistent with the results of the previous studies, among the three dimensions of AS, only social concern does not work (36).

The current research on mental health is very necessary, and it is necessary to continue to strengthen public mental health science publicity and psychological counseling during the COVID-19. All sectors of society should gradually start to prevent and respond to mental health problems after the pandemic, and strengthen the construction of the social psychological service system and improve the public's mental health development in the future.

## Limitations and Prospects

The current study investigated attention bias to negative information applied to the mediation effect of anxiety and depression, it still has several limitations. On the one hand, as a cross-sectional study, it is impossible to determine the causal relationship and direction between variables, and the self-reports cannot guarantee the objectivity and authenticity of the data. Future research can use longitudinal research to further study causality. On the other hand, besides the mediating effect of physical and cognitive concerns, there may also be some regulatory variables affecting the relationship between individual negative bias, anxiety and depression. Future studies may consider further exploring the relations of negative bias on mental health in more complex models.

## Conclusion

In sum, the current study found that negative bias was associated with levels of anxiety and depression, and physical and cognitive AS mediated associations between negative bias and anxiety and depression symptoms. The findings of this study provide theoretical support for intervention and guidance on individual mental health during the pandemic, and helps individuals increase their concern to negative emotions.

## Data Availability Statement

The raw data supporting the conclusions of this article will be made available by the authors, without undue reservation.

## Ethics Statement

The studies involving human participants were reviewed and approved by the Ethics Committee of Tianjin Normal University. The patients/participants provided their written informed consent to participate in this study.

## Author Contributions

SL designed the study protocol. XL conducted data collection, data management, cleaning, and analysis. SL and XL wrote the first draft of the paper. All authors contributed to the article and approved the submitted version.

## Funding

This research receives a grant from the Natural Science Foundation of China (Grant number 31800921).

## Conflict of Interest

The authors declare that the research was conducted in the absence of any commercial or financial relationships that could be construed as a potential conflict of interest.

## Publisher's Note

All claims expressed in this article are solely those of the authors and do not necessarily represent those of their affiliated organizations, or those of the publisher, the editors and the reviewers. Any product that may be evaluated in this article, or claim that may be made by its manufacturer, is not guaranteed or endorsed by the publisher.
